# Advancing vector biology research: a community survey for future directions, research applications and infrastructure requirements

**DOI:** 10.1080/20477724.2016.1211475

**Published:** 2016-06

**Authors:** Alain Kohl, Emilie Pondeville, Esther Schnettler, Andrea Crisanti, Clelia Supparo, George K. Christophides, Paul J. Kersey, Gareth L. Maslen, Willem Takken, Constantianus J. M. Koenraadt, Clelia F. Oliva, Núria Busquets, F. Xavier Abad, Anna-Bella Failloux, Elena A. Levashina, Anthony J. Wilson, Eva Veronesi, Maëlle Pichard, Sarah Arnaud Marsh, Frédéric Simard, Kenneth D. Vernick

**Affiliations:** 1 MRC-University of Glasgow Centre for Virus Research, Glasgow, UK; 2 Department of Life Sciences, Imperial College London, London, UK; 3 The European Molecular Biology Laboratory, The European Bioinformatics Institute, Wellcome Trust Genome Campus, Cambridge, UK; 4 Laboratory of Entomology, Wageningen University and Research Centre, Wageningen, The Netherlands; 5 Polo d'Innovazione di Genomica, Genetica e Biologia, Perugia, Italy; 6 Centre de Recerca en Sanitat Animal (CReSA), Institut de Recerca i Tecnologia Agroalimentàries (IRTA), Campus UAB, Barcelona, Spain; 7 Arboviruses and Insect Vectors Unit, Department of Virology, Institut Pasteur, Paris cedex 15, France; 8 Department of Vector Biology, Max-Planck-Institut für Infektionsbiologie, Campus Charité Mitte, Berlin, Germany; 9 Integrative Entomology Group, Vector-borne Viral Diseases Programme, The Pirbright Institute, Surrey, UK; 10 Swiss National Centre for Vector Entomology, Institute of Parasitology, University of Zürich, Zürich, Switzerland; 11 Department of Parasites and Insect Vectors, Institut Pasteur, Unit of Insect Vector Genetics and Genomics, Paris cedex 15, France; 12 MIVEGEC "Maladies Infectieuses et Vecteurs: Ecologie, Génétique, Evolution et Contrôle", UMR IRD224-CNRS5290-Université de Montpellier, Montpellier France; 13 CNRS Unit of Hosts, Vectors and Pathogens (URA3012), Institut Pasteur, Paris cedex 15, France

**Keywords:** Vector-borne diseases, Vector biology, Vector–pathogen interactions, Research infrastructures, Research requirements, Community survey

## Abstract

Vector-borne pathogens impact public health, animal production, and animal welfare. Research on arthropod vectors such as mosquitoes, ticks, sandflies, and midges which transmit pathogens to humans and economically important animals is crucial for development of new control measures that target transmission by the vector. While insecticides are an important part of this arsenal, appearance of resistance mechanisms is increasingly common. Novel tools for genetic manipulation of vectors, use of *Wolbachia* endosymbiotic bacteria, and other biological control mechanisms to prevent pathogen transmission have led to promising new intervention strategies, adding to strong interest in vector biology and genetics as well as vector–pathogen interactions. Vector research is therefore at a crucial juncture, and strategic decisions on future research directions and research infrastructure investment should be informed by the research community. A survey initiated by the European Horizon 2020 INFRAVEC-2 consortium set out to canvass priorities in the vector biology research community and to determine key activities that are needed for researchers to efficiently study vectors, vector-pathogen interactions, as well as access the structures and services that allow such activities to be carried out. We summarize the most important findings of the survey which in particular reflect the priorities of researchers in European countries, and which will be of use to stakeholders that include researchers, government, and research organizations.

## Graphical Abstract

**Figure:**
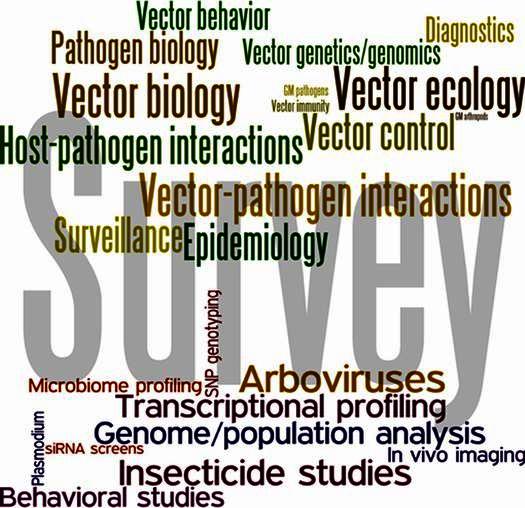

## Introduction

Vector-borne diseases (VBDs) such as malaria, dengue, and emerging threats such as chikungunya virus and Zika viruses have a major impact on human and animal health.^1–4^ While established technologies such as drugs, vaccines, and insecticides are likely to remain major components of control strategies against new vector-borne disease threats, issues such as pathogen and vector diversity [see, e.g. Ref.^5^], the challenges of vaccine and drug production^6–16^ including shortages (e.g. in the case of yellow fever virus vaccine, see *www.nc.cdc.gov/travel/news*-*announcements/yellow*-*fever*-*vaccine*-*shortage*-*2016*), resistance to drugs^17–19^, and insecticides [e.g. Ref.^20–26^] require that research into vector biology and control is continuously developed and strengthened. Finally, some vectors with mature reference genomes and toolkits, for example *Anopheles gambiae*, have become model research insects that now rival *Drosophila melanogaster* for questions on host–pathogen interactions, insect immunity, and population genomics.

Developments over the last decade including high-throughput genomics and transcriptomics [with corresponding data repositories and analysis tools such as VectorBase^27^], population genetics,^28–31^ improved methods for genetic manipulation of arthropods,^32–39^ studies on the influence of the mosquito midgut microbiome on pathogen transmission,^40–42^ investigations on the impact of the insect-specific viruses on arbovirus transmission,^43,44^ and the use of *Wolbachia* endosymbiotic bacteria that prevent pathogen transmission^32,45–48^ are suggesting promising new avenues of research into the control of vector-borne diseases. However, the specialized knowledge, cost, and infrastructure required to fully use new technologies can limit their dissemination and exploitation.

The European Union (EU) has identified access to specialized Research Infrastructures (RIs) offering unique research services to the international scientific community as a key to producing high quality science. RIs are defined as

Tools for science … RIs offer unique research services to users from different countries, attract young people to science, and help to shape scientific communities … RIs may be **‘**single-sited**’** (a single resource at a single location), **‘**distributed**’** (a network of distributed resources), or **‘**virtual**’** (the service is provided electronically)^49^

Such RIs can be research facilities, resources, and related services. Within the Framework Programmes (FP) of the EU, RI projects support the improvement of high-level facilities for research and allow access to the facilities by researchers in Europe and eligible member states. A wide range of research disciplines have been targeted by RI projects, including physics, information science, earth science, and medicine. RI projects are not research networks, but rather are tasked to identify the key unique and rare RI necessary for a research community and organize them so that researchers at institutes lacking necessary infrastructure can benefit from it in order to expand the scope of their research. Thus, RIs are exceptional facilities that permit experiments that could not routinely be done without this structure. Use of RI facilities by external researchers is provided as so-called Transnational Access (TNA), with access costs reimbursed by the RI project, thus provided at no cost to the end user.

One such RI project under the 7th EU Framework Programme (FP7) was INFRAVEC, which focused on developing and providing research resources for insect vector biology from 2009 to 2014. INFRAVEC, which was constituted as an EC Starting Community under FP7, obtained the opportunity to continue as an advanced community (AC) called INFRAVEC-2 under the Horizon 2020 framework. Conditions have changed since the inception of the FP7 INFRAVEC project, including the emergence and transmission of arboviruses in Europe and elsewhere, as well as widening the project scope to include vector-borne diseases of economically important animals and the most recently developed innovative technologies. Collecting updated information about the current and anticipated future infrastructure needs of the vector biology research community and other stakeholders is an important step to ensure that the services offered via TNA reflect actual needs of the advanced community. To our knowledge, there is no comparable recent source of such vector research community-needs information. Here, we present the findings of a survey of scientists and associated stakeholders in the field of vector biology or fields that are linked to vector biology such as pathogen studies, which will help to define priorities and requirements within INFRAVEC-2 but should also be of interest to governments, research organizations, and researchers in the field. Participation numbers suggest that in particular European, research priorities are reflected in the results, but the data can inform stakeholders worldwide.

## Materials and methods

### Survey structure

A questionnaire (S1 Table) was sent to organizational email lists (European Society for Vector Ecology; the journal Pathogens and Global Health; National Center of Expertise in Vectors (CNEV, France); CIRM-Italian Malaria Network; FP7 INFRAVEC mail list; International Meeting on Arboviruses and their Vectors mail list; BioInsectes; EU/DEVCO MEDILABSECURE network; WHO vector control working group), as well as to other lists available to the authors (institutional mailing lists, etc.). The questionnaire was sent as a URL link to the online form along with an explanatory note to scientists in the vector biology field and associated stakeholders. The questionnaire request was spontaneously retransmitted by an unknown number of recipients to organizational and other lists. Although not quantifiable, the degree of retransmission suggests that exposure of the research community to the survey was high.

Briefly, the cover note explained the aims of the INFRAVEC-2 community, followed by a series of questions. The key areas covered by the survey are as follows: (1) vectors and vector-borne pathogens studied by survey participants, (2) research area (with several responses allowed), (3) infrastructures available at the respondents home institution including those for vector and animal research, (4) ease of access to vector research facilities outside the survey participants’ home institution, (5) infrastructures that participants would use, offered by the facilities at no cost to user, (6) identification of research priorities over the next 5–10 years, and (7) additional feedback. The survey was carried out from October to November 2015. Respondents were given the opportunity to provide their name and institution, although this was not required for completion of the questionnaire. However, all respondents (*n* = 211) identified themselves, indicating that repeat voting or vote stuffing is not a concern for interpretation of the results. All results shown here are anonymized, and no survey participant details published.

## Results and discussion

In total, 211 responses were obtained (see S2 Table). Approximately 88% of respondents were from countries across Europe, with France, and then the UK providing the highest numbers of responses. This suggests that the results reflect a good overview of current priorities in European vector biology and vector research areas. Below we summarize and analyze the data obtained in the survey.

### Research areas: arthropods and pathogens relevant to survey participants

Our goal was to obtain an overview of the research areas and work of survey participants, which are thus likely to guide their future research needs (S1 Table, Survey Questionnaire). First, respondents indicated vectors relevant to their research as major or minor area of interest (Table 1). *Aedes* species mosquitoes were the top field, followed by *Anopheles* and *Culex* species. The strong interest in aedine species may reflect the emergence of arboviruses such as chikungunya transmitted by *Ae. aegypti* and *Ae. albopictus* as well as the expansion of the latter species in Europe (and other areas) and acting as arbovirus vector.^50–56^ Despite their importance in the European context as major vectors of pathogens, comparatively little research is carried out on ticks that transmit viruses including tick-borne encephalitis virus and Crimean-Congo hemorrhagic fever virus)^57,58^ and bacteria such as *Borrelia*.^59,60^ However, some recent studies on tick-borne pathogens in ticks or tick cells^61–67^ show that a knowledge base is present in Europe that can be built on. The same is true for *Culicoides* midges that transmit the emerging viruses Schmallenberg and bluetongue and thus became vectors of arboviruses in Europe.^68–70^ Indeed, studies on midge biology are carried out only in few places with specialist resources and expertise such as the Pirbright Institute, most likely because *Culicoides*-borne pathogens have only recently emerged within the EU, and thus, there has been little incentive or resources to develop and maintain the skills and infrastructure needed for such research, such as laboratory colonies of *Culicoides*. This suggests that the European vector biology community has some expertise but presently lacks sufficient opportunities and resources to expand research on these vectors. Among the category ‘Other’, comments by participants indicated phlebotomines/sand flies as a key area, with tsetse flies, fleas, triatomines, and tabanids/horse flies also mentioned.

**Table 1:** Research areas and interests of the survey participants. Numbers of responses are indicated as Major or Minor depending on vector listed, or in the category ‘Other’ which incorporates other vectors not specifically listed (selection of responses shown)

Arthropod	Major	Minor
*Aedes* spec.	102	38
*Culex* spec.	66	46
*Anopheles* spec.	79	42
*Culicoides* spec.	24	32
Ticks	55	44
Other	42	49

Notes:

Vectors mentioned under ‘Other’ (selection of most mentioned): phlebotomines/sandflies^33^, fleas,^13^ tsetse flies,^7^ triatomines,^4^ tabanids/horse flies.^6^

We also quantified the major and minor interests of survey participants (Table 2). There was a notably strong indication of research interests in arboviruses, mainly affecting humans but livestock as well. These research interests and activities are likely due to the emergence and importance of arboviruses such as chikungunya, Zika, Schmallenberg, and bluetongue.^2,51,68,70,71^ Given the historically important role of malaria research also in Europe (though much research is conducted overseas in affected areas, e.g. in Africa, often by groups working overseas and/or in collaboration with local teams), the overall importance in the vector field is not surprising. Of note was the impact of tick-borne pathogens in the category ‘Other’, and this is worth mentioning, especially with the impact of Lyme disease across Europe and North America^72^ and surge in interest in Crimean-Congo hemorrhagic fever virus.^73,74^

**Table 2:** Pathogens relevant to the survey participants. Numbers of responses are indicated as Major or Minor depending on pathogen category listed, or in the category ‘Other’ which incorporates other pathogens not specifically listed (selection of responses shown)

Pathogen category	Major	Minor
Arboviruses, human	96	40
Arboviruses, livestock	44	45
*Plasmodium* spec.	66	31
Other	68	28

Pathogens mentioned under ‘Other’ (selection of most mentioned): *Leishmania*,^15^ trypanosomes,^8^ tick-borne pathogens^23^

To describe their activities in more detail, we collected further data on the research areas of interest to the survey participants (Table 3). In general, vector biology describes the research of over half of the participants; however, this is a very broad term. Vector ecology, behavior, and control were also commonly reported. Of note, genetic modification and vector immunity remain relatively small fields despite important advances in these areas; these include CRISPR-Cas9-mediated genetic modification of mosquitoes^75,76^ and also deeper understanding of vector anti-pathogen responses.^41,77–80^ Interest may increase with better tools and access to new resources such as strains and facilities. The survey data showed that studies of pathogens either directly or within the context of host-pathogen or vector–pathogen interactions are a key area of research. This needs to be emphasized as it integrates disciplines such as virology, parasitology, cell biology, microbiology, and genetics into the vector field. Similarly, surveillance, diagnostics, and epidemiology were important areas and this (alongside vector control, behavior, and ecology) was an indication of the applied character of many activities in the field of vector-borne diseases.

**Table 3:** Details of research areas relevant to survey participants. Numbers of responses are shown by research area, or in the category ‘Other’ which incorporates fields not specifically listed (selection of responses shown)

Research area	Response counts	Research area	Response counts
Vector biology	119	Host–pathogen interactions	102
Vector genetics/genomics	68	Vector–pathogen interactions	116
Vector immunity	32	Epidemiology	99
Vector behavior	77	Surveillance	96
Vector ecology	117	Diagnostics	69
Vector control	98	Other	29
Genetically modified arthropods	20		
Pathogen biology	88		
Genetically modified pathogens	28		

Other: evolution/population genetics, insecticide, etc. (few precise indications given).

### Assessment of currently available facilities

Knowledge of availability and/or ease of access to research infrastructures is a key factor in the future planning of research activities. Survey participants were therefore asked to indicate their current organization’s current capabilities. As shown in Table 4, survey participants indicated a certain level of capacity to provide vectors but also material across the community. Moreover, facilities for biosafety level (BSL) 2 and 3 experiments with vectors, animals, and pathogens are available in several places. The concept of RI can be extended to reagent provision and has been successfully established by FP7 INFRAVEC and the European Virus Archive (http://www.european-virus-archive.com). This indicates an existing infrastructure base that can be developed and made available for research on vectors and pathogens on a wider basis (e.g. those who do not have immediate access to BSL 3 level insectaries but would require experiments to be carried out in such facilities) through communities such as INFRAVEC-2.

**Table 4:** Research infrastructures and resources available to survey participants. Various types of structures relevant to vector and pathogen research are indicated

Available facilities and resources	Response counts
Furnish vectors to external users	74
Furnish BSL2/BSL3 infected vectors/extracts to external users	32
BSL2 containment: arthropod infections	91
BSL3 containment: arthropod infections	60
Pathogen work in cell culture	128
BSL2 or BSL3 containment: small animal work	83
BSL2 or BSL3 containment: large animal work	27

### Assessment of infrastructure and service requirements

When survey participants were asked to indicate how many had requested access to insectaries at BSL2 or 3 in other institutions, in total 62 positive responses were received. However, of these, 18 responses indicated that access could not be granted in a timely manner. This suggests that inability to consistently access secure insectary facilities comprises a systematic weakness that impedes research on vector–pathogen interactions and may also explain the weaker interest in vector immunity studies, for example. The relevant secure insectary facilities exist in Europe (Table 4), and thus, a mutualized network of insectaries at BSL2 and 3 could resolve access limitations and promote elevated levels of vector research under BSL2 and 3 conditions.

Access needs or provision of infected vectors or extracts from infected vectors were assessed, and participants were asked to indicate which pathogens or facilities/services would be of interest in the context of INRFAVEC-2 where these are free of cost (or the requirement for collaboration) for the end user (Table 5). Although the questions below were originally aimed at potential European users, all answers were taken into account. Survey data show that in particular services and structures for arbovirus research would likely generate strong demand. Again this may be due to the surge in research in this field described above. Similarly, BSL2 and 3 studies on infected vectors and insecticides as well as behavior scored highly. Regarding technologies novel for the field, functional siRNA screens, and imaging of vectors did not score particularly high but this demand may increase in the future, particularly if facilities were available for access.

**Table 5:** Infrastructure services (vector infection and vector–pathogen interactions) for the vector research community. Survey participants responded whether the services listed here (vector infection and vector–pathogen interactions) to study vector infections and vector–pathogen interactions would be of use if offered free of cost. Response counts are grouped into Likely, Not likely, or Possible use of the infrastructure/service

Infrastructure/service: vector infection and vector–pathogen interactions	Likely	Not likely	Possible
Arboviruses	90	46	31
*Plasmodium falciparum*	36	77	33
Infected vector and insecticide studies	83	38	50
Behavioral studies with infected vectors	64	50	42
*In vivo* imaging with infected vectors	48	58	41
Functional siRNA screens of vector cells	35	68	36
Other needs	21	39	15

Category ‘Other needs’ included various *Plasmodium* species, *Leishmania*, tsetse flies, etc.

Vector genetics and genomics [see www.vectorbase.org,^27^] but also studies of vector microbiomes [given their influence on mosquito infection with arboviruses and parasites^40–42,81^] are expanding fields. These research areas have strongly benefited from high-throughput sequencing techniques and bioinformatics. Survey participants were enthusiastic about developing insect vector-oriented infrastructures, services, and expertise in high-throughput genomics and bioinformatics, especially transcriptional profiling and genome and population analysis (Table 6).

**Table 6:** Infrastructure services (vector genomics and bioinformatics) for the vector research community. Survey participants responded whether the services listed here (vector genomics and bioinformatics) would be of use if offered free of cost. Response counts are grouped into Likely, Not likely, or Possible use of the infrastructure/service

Infrastructure/service: vector genomics and bioinformatics	Likely	Not likely	Possible
Transcriptional profiling	75	42	53
Genome or population analysis	72	43	54
Bacterial microbiome profiling	45	63	41
Population or focused SNP genotyping	39	63	48
Other needs	10	41	8

Category ‘Other needs’ included proteomics, metabolomics.

The era of genomics has brought about much needed information on vector genomes [see, e.g. Ref.^82–84^]. Genetic manipulation of genomes in basic biological studies of gene/sequence structure and function and applications based on genome manipulation [see, e.g. Ref.^85–87^] are useful tools to maximize the value of this information, and for example, CRISPR-Cas9-mediated genome manipulation is an important technical advance also for the vector field.^75,88^ We therefore asked survey participants about their interest in applying genome editing technologies within their work. As shown in Table 7, there was particularly strong interest in genetic manipulation of aedine mosquitoes. *Culicoides* midges seemed at present a less popular subject, probably at least in part because the community is small as mentioned above, as well as that the technologies have not yet been applied to this system or general issues with establishing colonies of important midge vector species. Among the category ‘Other’, ticks stood out revealing a need to establish infrastructures for tick research.

**Table 7:** Infrastructure services (vector genome editing) for the vector research community. Survey participants responded whether vector genome editing would be of use if offered free of cost. Response counts are grouped into Likely, Not likely, or Possible use of the infrastructure/service

Infrastructure/service: vector genome editing	Likely	Not likely	Possible
*Anopheles* spec.	35	71	44
*Aedes* spec.	60	60	33
*Culicoides* spec.	20	85	17
Other	27	53	7

‘Other’ included ticks,^17^ phlebotomines,^5^ *Culex* spec., and tsetse flies (both 4).

Studies on vectors (infected, uninfected, or genetically modified) often include components that analyze behavior and ecology. A further section of this survey therefore focused on a number of specific potential requirements in this area. As indicated in Table 8, the interest to work in field sites in endemic countries if access could be provided as well as standardized behavioral assays and bioassays for vectors generated strong positive responses. This suggested a need for these in the vector research community. Positive responses for large cage studies (controlled indoors or semi-controlled outdoors) were also strong considering that such applications are very specialized, and the facilities are rare. However, this illustrates the potential contribution of a RI project, because community mutualization of rare infrastructures can allow access to state-of-the-art facilities for researchers with occasional needs. In the future, the possibility to access such facilities may become stronger as more genetically modified vectors will be assessed in pre-release assays. Few positive responses for electrophysiology experiments were obtained, suggesting that there is no major need for additional facilities beyond what is already in place.

**Table 8:** Infrastructure services (vector ecology and behavior) for the vector research community. Survey participants responded whether specific services or infrastructures to study vector ecology and behavior would be of use if offered free of cost. Response counts are grouped into Likely, Not likely, or Possible use of the infrastructure/service

Infrastructure/service: vector ecology and behavior	Likely	Not likely	Possible
Facilitated work at endemic country field sites	96	39	43
Electrophysiology	14	99	23
Standardized vector behavioral assays and bioassays	65	52	52
Large cage studies (controlled large indoor insectary)	64	61	46
Large cage studies (semi-controlled outdoor large cages)	46	77	40
Other needs	7	44	3

Very few responses to ‘Other needs’ given, for example, cage trials in Europe.

Survey participants were also asked about their requirements for more specific vector-related data and research resources such as reference genomes, specific cell lines, and mosquito strains (Table 9). Results indicated that in particular, a bank of standard vector colonies would be of interest to the community. Easily accessible quality-controlled vector colonies available from a European repository could be an important influence promoting comparability and reproducibility of experimental infections and other results across laboratories. Such newly initiated colonies would not suffer from some of the weaknesses of current colonies that are widely used by default, including bottlenecking and loss of genetic diversity, long-term adaptation to fitness under insectary conditions with unknown consequences for traits related to vector competence, and even admixture of multiple species within the colony. New, rationally initiated colonies can for example be characterized for defined parameters such as competence for a given pathogen, defined genome sequences, and physiological properties to allow experiments to be better controlled. Similarly, vector systematics and collections generated high interest. However, the practices of systematics may be at a juncture, because the technological capacity will soon be available to whole-genome sequence large numbers of unidentified individuals of a putative vector clade, and cluster them bioinformatically to determine phylogenetic relatedness. These results will need to be compared to existing collections, including voucher specimens. Perhaps surprisingly, new reference and cloned vector cell lines did not score highly but these may be of interest to smaller research areas such as virologists who carry out particular types of studies. Cell lines may be of less interest in malaria vector research where the biology is not consistent with simple cell models of *Plasmodium*–mosquito interaction. Despite high interest in *Wolbachia* to block pathogen transmission,^47^ generation of novel trans-infected vector strains was also not a priority. Finally, a small number of responses under ‘Other needs’ mentioned the importance of training.

**Table 9:** Infrastructure services (vector biology resources) for the vector research community. Survey participants responded whether specific resources for vector biology would be of use if offered free of cost. Response counts are grouped into Likely, Not likely, or Possible use of the infrastructure/service

Infrastructure/service: vector biology resources	Likely	Not likely	Possible
Bank of standard vector reference strains (genome and RNA sequenced)	85	34	54
Colonization of novel vector strains and species	76	45	53
Production of new reference vector cell lines (genome & RNA sequenced)	38	74	38
Production of cloned vector cell lines	39	75	37
Production of microbiome-free mosquitoes	28	82	40
*Wolbachia*-trans-infected vector strains	23	76	52
Vector systematics and collections	62	52	55
Other needs	5	44	3

Very few responses to ‘Other needs’ given, mainly mentioning training needs.

Our survey specifically addressed training needs, community networking, and communication. As shown in Table 10, all suggestions – training in vector BSL2 and 3 methods, training in bioinformatics and genomics, and scientific communication by conferencing – were positively received by the survey participants. Clearly these are areas of need that should be developed as a real requirement within the vector research field.

**Table 10:** Infrastructure services (training and networking activities) for the vector research community. Survey participants responded whether specific services or infrastructures in the areas of training and networking activities would be of use if offered free of cost. Response counts are grouped into Likely, Not likely, or Possible use of the infrastructure/service

Infrastructure/service: training and networking activities	Likely	Not likely	Possible
Training in BSL2 and 3 vector infection and study techniques	96	30	51
Training in bioinformatics and genomic analysis	107	25	53
Conferencing	102	16	59
Other needs	8	32	7

Very few responses to ‘Other needs’ given; one example: training of field workers and students in field identification.

Survey participants were also asked to give their opinions in a text field on research priorities for vector biology over the next 5–10 years. Answers varied but some key areas were identified: (1) Vector interactions with hosts and pathogens, including vector competence and transmission; (2) insecticide resistance and novel insecticides; (3) ecology and behavior, including of infected vectors, introduction of vectors, and mathematical modeling approaches to such questions, etc.; (4) vector control, novel control measures, and surveillance; (5) vaccines, including anti-vector vaccines; (6) vector genomics/genetics and bioinformatics. Although no survey can be complete, the data presented here yield a valuable qualitative picture of the needs and requirements in disease vector biology, especially of European scientists. It is clear that this survey cannot give a fully representative image of a wide and diverse community. The email lists which were used to contact potential respondents initially reflect activities in which the authors are involved in; however, the authors represent also a cross section of scientists involved in this research area. We thus expect this study to be relevant to stakeholders such as governments, research councils, and organizations but also researchers as priorities for future activities such as those planned by INFRAVEC-2 are determined.

## Supplemental data

The supplementary material for this paper is available online at http://dx.doi.10.1080/20477724.2016.1211475.

## Funding

This work was supported by UK Medical Research Council [grant number MC_UU_12014]; European Research Council [grant number #323173 AnoPath]; European Commission and French Laboratoire d’Excellence [grant number #ANR-10-LABX-62-IBEID]; UK Biotechnology and Biological Sciences Research Council [grant number BBS/E/I/00002066].

## Supplementary Material

YPGH_1211475_Supplementary_Material.zip
