# Supplementary material for: Advancing vector biology research: a community survey for future directions, research applications and infrastructure requirements
Source: Pathog Glob Health. 2016 Jun;110(4-5):164–72. doi: 10.1080/20477724.2016.1211475 (PMC5072118; doi:10.1080/20477724.2016.1211475)
Supplement: YPGH_1211475_Supplementary_Material.zip [file YPGH_A_1211475_SM7122.zip › YPGH_1211475_Supplementary_Material/S1 Table.pdf]

HORIZON 2020 INFRAVEC-2 Questionnaire

Introduction

A consortium of European institutions based in the former FP7/INFRAVEC project is responding to the new H2020 call “Integrating Activities for Advanced Communities” of the European Research Infrastructures (RI) Programme under the item “Research Infrastructures for the control of vector-borne diseases”. The primary purpose of an “integrated infrastructure” is to provide the EU scientific community with access to its network of RI facilities and services, without charge to the end user, at the state-of-the art premises of participating institutions. Access to the specialized RI enables European researchers and SME to carry out experiments beyond their current capacities.

Building upon the major achievements of FP7/INFRAVEC in forging a European Starting Community of insect vector RI, we worked with EC representatives to generate the current H2020 call for an Advanced Community (AC). With strong commitment obtained from collaborating institutions hosting top-level specialized EU facilities for vector-borne disease (including Institut Pasteur FR, Imperial College UK, Centre de Recerca en Sanitat Animal (IRTA-CReSA) ES, Wageningen University NL, University of Glasgow UK, Institut de Recherche pour le Développement (IRD)-Montpellier FR, Polo d'Innovazione di Genomica, Genetica e Biologia (Polo GGB) IT, Pirbright Institute UK, Max-Planck-Institut für Infektionsbiologie DE, Radboud University Medical Center NL, and EMBL European Bioinformatics Institute DE), the group, chaired by K. Vernick (Institut Pasteur), has been invited to organize the AC.

The RI consortium will provide enabling infrastructures and support for research on disease vectors and their pathogens. We have listed possible RI and services within the following questionnaire, and we would like to solicit as wide as possible feedback from potential users in order to understand the major needs of the vector biology community. Your particular requirements and feedback will have strong impact on how the project will be structured, as this “integrated infrastructure” needs to be tightly tailored to, and inspired by real community needs. Please, take a minute to fill in a short questionnaire (~15 min) that will help us mobilize necessary resources for the future of our community.

Please feel free to forward this email to relevant colleagues. The primary target audience is EU insect vector researchers and SME, but we welcome replies from outside the EU as well. All individual replies and identity information will be kept confidential. Questions can be addressed to email: infravec-survey@pasteur.fr

Please complete this form as soon as you can. The survey will close November 25th 2015.

Thank you for your collaboration.

Q1. Please provide your name and contact details.

Title

First name

Last name

Position

Organization

Country

Email (optional)

Providing an email address is optional, but will permit us to keep you informed about the consortium; contact information will not be shared with other parties.

Q2. Please identify the arthropod vectors and/or vector borne pathogens that you research

Select all that apply

	MAJOR	Minor
Aedes		
Culex		
Anopheles		
Culicoides		
Ticks		
Other		

If other other area

Please specify below

Select all that apply

	MAJOR	minor
Arboviruses (human)		
Arboviruses (livestock)		
Plasmodium		
Other		

If other other area
Please specify below

Q3. Please select the most relevant areas that describe your research interests from the list below

Select all that apply

- ☐ Vector biology
- ☐ Vector genetics/genomics
- ☐ Vector immunity
- ☐ Vector behavior
- ☐ Vector ecology
- ☐ Vector control
- ☐ Genetically modified arthropods

- ☐ Pathogen biology
- ☐ Genetically modified pathogens

- ☐ Host-pathogen interactions
- ☐ Vector-pathogen interactions
- ☐ Epidemiology
- ☐ Surveillance
- ☐ Diagnostics
- ☐ Other

If other
Please specify below

Q4. Does your organization have infrastructure facilities described by the list below?

Select all that apply

- [ ] To rear arthropod vectors
- [ ] To furnish vectors as a provider to external users
- [ ] To infect arthropods in BSL-2 containment
- [ ] To infect arthropods in BSL-3 containment
- [ ] To work with pathogens using in vitro cell cultures
- [ ] To infect small animals under BSL-2 or 3 containment
- [ ] To infect large animals under BSL-2 or 3 containment
- [ ] To furnish BSL-2 or -3 infected vectors or extracts to external users

Q5. Have you ever tried to access BSL-2 or 3 vector research facilities based at organizations other than your own?

- ( ) Yes
- ( ) No
- ( ) Not applicable

a. If yes, did the facility have sufficient capacity to accommodate your request in a timely manner?

- ( ) Yes
- ( ) No

Q6. Which infrastructure services offered to European users would you be likely to use, with user access costs paid by a Horizon 2020 Research Infrastructure consortium (i.e., at no charge to the end-user). Items provided as user access or custom service, which does not require scientific collaboration with the providing facility.

a. VECTOR INFECTION AND VECTOR-PATHOGEN INTERACTIONS. Access to BSL-2 or 3 secure insectary facilities for infection of vectors, or provision of infected vectors or extracts custom-generated by such a facility. Vectors infected by the following pathogens, and for the following research purposes (select all that apply).

	Likely	Possible	Not likely
1. Arboviruses			
2. Plasmodium falciparum			
3. Infected vectors and insecticide studies			
4. Behavioral studies with infected vectors (e.g., odorant/host choice)			
5. In vivo imaging of infected vectors (e.g., confocal, spinning disk)			
6. siRNA functional screening of vector cell lines			
7. Other needs			

If other needs--please specify below

Q6. Which infrastructure services offered to European users would you be likely to use, with user access costs paid by a Horizon 2020 Research Infrastructure consortium (i.e., at no charge to the end-user). Items provided as user access or custom service, which does not require scientific collaboration with the providing facility.

b. VECTOR GENOMICS AND BIOINFORMATICS. High-throughput genomic services. If desired, with upstream bioinformatic design advice and downstream bioinformatic analysis (select all that apply)

	Likely	Possible	Not likely
1. Transcriptional profiling by Illumina RNA-seq			
2. Genome or population analysis by Illumina DNA sequencing			
3. Bacterial microbiome profiling by 16S rRNA amplicon deep sequencing			
4. Population or focused SNPgenotyping (e.g., Sequenom)			
5. Other needs			

If other needs--please specify below

Q6. Which infrastructure services offered to European users would you be likely to use, with user access costs paid by a Horizon 2020 Research Infrastructure consortium (i.e., at no charge to the end-user). Items provided as user access or custom service, which does not require scientific collaboration with the providing facility.

c. VECTOR GENOME EDITING. Provision of custom genetic modification of your requested target gene or sequence using CRISPR or other technology in vectors (select all that apply). Could also include phenotyping the mutation effect by pathogen challenge under (a) above.

	Likely	Possible	Not likely	
1. Anopheles				
2. Aedes				
3. Culicoides				
4. Other needs				

If other needs--please specify below

Q6. Which infrastructure services offered to European users would you be likely to use, with user access costs paid by a Horizon 2020 Research Infrastructure consortium (i.e., at no charge to the end-user). Items provided as user access or custom service, which does not require scientific collaboration with the providing facility.

d. VECTOR ECOLOGY AND BEHAVIOR. Provision of access to facilities or custom-performed assays (select all that apply). Could also include pathogen infection of vectors under (a) above.

	Likely	Possible	Not likely
1. Facilitated work at endemic country field sites, Africa, Asia, S. America (population & epidemiology studies)			
2. Electrophysiology / EAG			
3. Standardized vector behavioral tests & bioassays (e.g. odorant, host choice)			
4a. Large-cage studies (e.g. behavior, fitness,reproduction, test of modified genetic strains) in completely controlled indoor large insectary			
4b. Large-cage studies (e.g. behavior, fitness,reproduction, test of modified genetic strains) in semi-controlled outdoor large cages (Africa)			
5. Other needs			

If other needs--please specify below

Q6. Which infrastructure services offered to European users would you be likely to use, with user access costs paid by a Horizon 2020 Research Infrastructure consortium (i.e., at no charge to the end-user). Items provided as user access or custom service, which does not require scientific collaboration with the providing facility.

e. VECTOR BIOLOGY RESOURCES. Provision of vector research resources by request (select all that apply).

	Likely	Possible	Not likely
1. Bank of standard vector reference strains (genome & RNA sequenced)			
2. Colonization of novel vector strains and species			
3. Production of new reference vector cell lines (genome & RNA sequenced)			
4. Production of cloned vector cell lines			
5. Production of microbiome-free mosquitoes			
6. Wolbachia transinfected vector strains			
7. Vector systematics and collections			
8. Other needs			

If other needs--please specify below

Q6. Which infrastructure services offered to European users would you be likely to use, with user access costs paid by a Horizon 2020 Research Infrastructure consortium (i.e., at no charge to the end-user). Items provided as user access or custom service, which does not require scientific collaboration with the providing facility.

f. TRAINING AND NETWORKING ACTIVITIES. Promotion of expertise using standardized, comparable practices, scientific exchange.

	Likely	Possible	Not likely
1. Training in BSL-2 and 3 vector infection and study techniques			
2. Training in bioinformatics and genomic analysis			
3. Conferencing			
4. Other needs			

If other needs--please specify below

Q7. In your opinion, what are the top research priorities (up to 5) in vector biology and/or vector borne disease that need to be addressed in the next 5-10 yrs in the European research context?

1.

2.

3.

4.

5.

Thank you for participating!

Please use the space provided below to send us any additional feedback on this survey.

Google Forms
