# Supplementary material for: Advancing vector biology research: a community survey for future directions, research applications and infrastructure requirements
Source: Pathog Glob Health. 2016 Jun;110(4-5):164–72. doi: 10.1080/20477724.2016.1211475 (PMC5072118; doi:10.1080/20477724.2016.1211475)
Supplement: YPGH_1211475_Supplementary_Material.zip [file YPGH_A_1211475_SM7122.zip › YPGH_1211475_Supplementary_Material/S2 Table.pdf]

| | Participants/country |
|---|---|
| **Europe** | |
| Albania | 2 |
| Austria | 2 |
| Belgium | 4 |
| Bosnia-Herzegovina | 1 |
| Bulgaria | 2 |
| Croatia | 1 |
| Czech Republic | 3 |
| Denmark | 1 |
| Estonia | 2 |
| Finland | 1 |
| France | 56 |
| Germany | 11 |
| Greece | 3 |
| Hungary | 1 |
| Italy | 15 |
| Kosovo | 1 |
| Latvia | 1 |
| Luxembourg | 2 |
| Moldova | 1 |
| Macedonia | 2 |
| Montenegro | 1 |
| Portugal | 8 |
| Romania | 2 |
| Serbia | 4 |
| Slovakia | 2 |
| Slovenia | 2 |
| Spain | 15 |
| Sweden | 4 |
| Switzerland | 3 |
| The Netherlands | 4 |
| UK | 28 |
| **Asia** | |
| Armenia | 1 |
| Burkina Faso | 2 |
| Cambodia | 1 |
| Israel | 2 |
| Palestine | 2 |
| Singapore | 1 |
| Turkey | 3 |
| **Africa** | |
| Algeria | 1 |
| Cameroon | 1 |
| Egypt | 1 |
| Morocco | 2 |
| South Africa | 1 |

| | |
|---|---|
| **Australia** | |
| Australia | 1 |
| **North America** | |
| USA | 7 |
